# Aneurysmal bone cyst of the mandible: A case report and review of literature

**DOI:** 10.4103/0973-029X.80014

**Published:** 2011

**Authors:** Parvathi Devi, VB Thimmarasa, Vishal Mehrotra, Mayuri Agarwal

**Affiliations:** *Department of Oral Medicine and Radiology, Rama Dental College, Hospital and Research Centre, A 1/8, Lakhanpur, Kanpur, India*

**Keywords:** Aneurysmal bone cyst, psuedocyst, benign bone lesions, mandible

## Abstract

Aneurysmal bone cyst (ABC) is rare benign lesions of bone which are infrequent in craniofacial skeleton. ABC’s are characterized by rapid growth pattern with resultant bony expansion and facial asymmetry. We describe a case of ABC in a 25 year old male patient affecting the body of the mandible with expansion and thinning of the buccal and lingual cortical plates. Treatment consisted of surgical curettage of the lesion. A one year follow- up showed restoration of facial symmetry and complete healing of the involved site.

## INTRODUCTION

Aneurysmal bone cyst (ABC) has been recognized since 1893 when it was described as an ossifying hematoma by Van Arsdale.[1] Jaffe and Lichtenstein were the first to recognize ABC as an intraosseous, osteolytic lesion, chiefly affecting the metaphyseal region of long bones and vertebrae. Bernier and Bhaskar described the first case of ABC in the jaws in 1958.[2,3]

ABC is a benign cystic lesion of bone, composed of blood-filled spaces separated by connective tissue septa containing fibroblasts, osteoclast-type giant cells and reactive woven bone.[4] Fifty percent of ABCs arise in the long bones and 20% in the vertebral column. It accounts for 1.5% of the nonodontogenic, nonepithelial cysts of the mandible.[1,5] It is found more frequently in the mandible than the maxilla (3:1) with preponderance for the body, ramus and angle of the mandible. It affects young persons under 20 years of age with no gender predilection.[5,6]

ABC can be classified into three types. Conventional or vascular type (95%) manifests as a rapidly growing, expansive, destructive lesion causing cortical perforation and soft tissue invasion. The solid type (5%) may present as a small asymptomatic lesion first noticed as radiolucency on a routine radiograph or as a small swelling.[7,8] A third form or mixed variant demonstrates features of both the vascular and solid types. It may be a transitory phase of the lesion because sudden activation or rapid enlargement of stable lesions has been reported.[8]

## CASE REPORT

A 25-year-old male patient reported to the Department of Oral Medicine and Radiology with a complaint of an asymptomatic swelling in the right lower back teeth region since 1 year, which had gradually increased to the present size. His medical and family history was unremarkable and there was no history of trauma. On extraoral examination, facial asymmetry was apparent with a diffuse swelling involving the right side of the lower jaw, measuring approximately 3 × 1 cm. The swelling was firm and nontender. Intraoral examination revealed a diffuse swelling in relation to right lower second premolar, first, second and third molars with vestibular obliteration. There was expansion of buccal and lingual cortical bone. On aspiration, blood-tinged fluid was obtained and electrical pulp testing showed that the involved teeth were nonvital.

**Figure 1:**
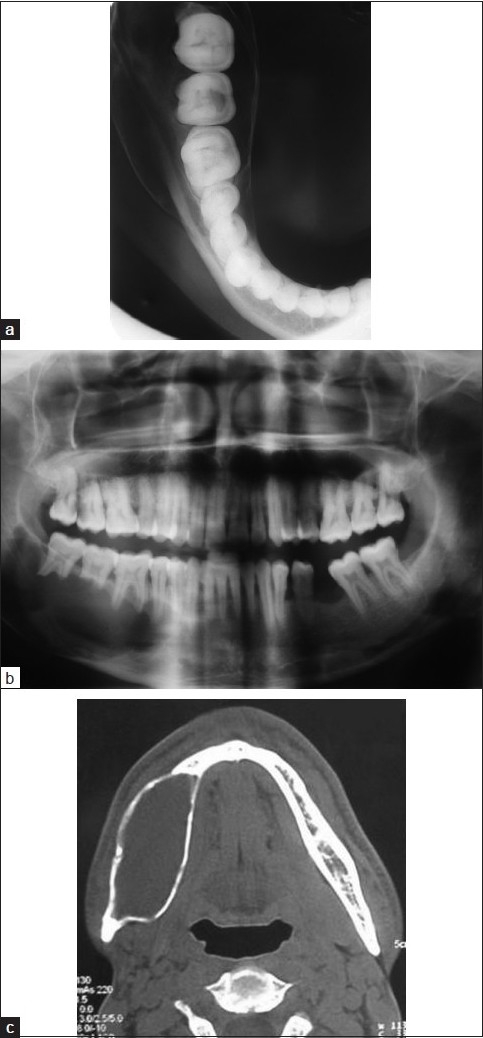
(a) Mandibular occlusal radiograph showing expansion of the cortical plates; (b) panoramic radiography showing a unilocular radiolucency extending from 45 to 48 region; (c) axial CT image of the mandible showing cortical expansion and thinning

**Figure 2:**
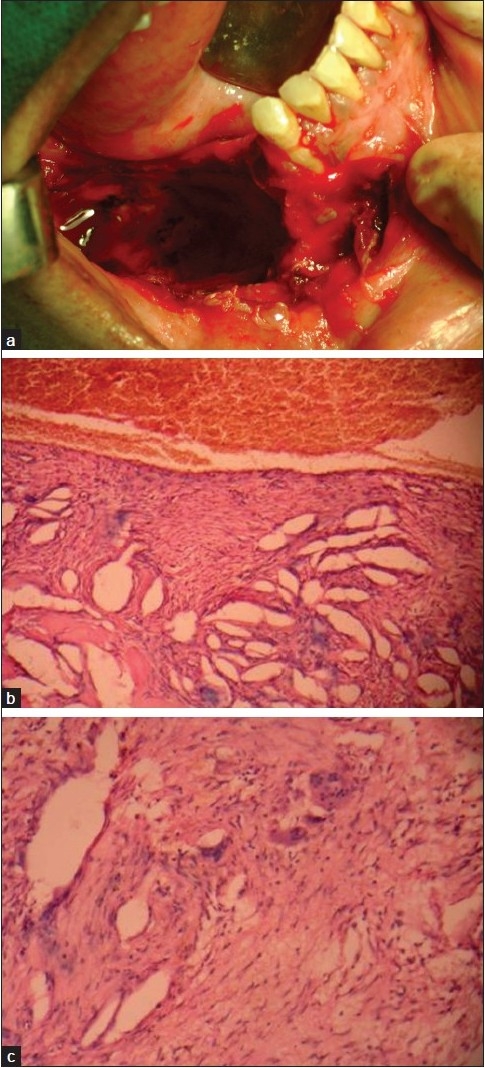
(a) Intraoperative photograph after the curettage; (b) photomicrograph of the lesion showing numerous vascular spaces lined by endothelial cells and multinucleated giant cells (H and E, 10×); (c) photomicrograph showing multinucleated giant cells in the fibrous tissue (H and E, 40×)

Mandibular occlusal radiograph showed expansion of the cortical plates [Figure 1a] and a panoramic radiograph revealed a large unilocular radiolucency present in the body of mandible, extending from the root of right second premolar to the retromolar region. There was thinning but no discontinuity of the lower border of the mandible and displacement of the mandibular canal toward the lower border of mandible and radicular resorption of involved teeth [Figure 1b]. Axial computed tomographic examination was performed and revealed a well-circumscribed bony expansile cystic lesion measuring 6.1 × 2.5 × 4 cm in size, with the epicenter at ramus of mandible and extending into right half of body of mandible [Figure 1c]. The involved teeth were removed and curettage of the lesion was performed under general anesthesia [Figure 2a] and the tissue was sent for histopathologic evaluation. The microscopic examination revealed numerous small and large vascular spaces lined by endothelial cells. Abundant pools of RBCs were seen. Hemosiderin pigment was seen at places along with giant cells, which was suggestive of ABC [Figure 2b and 2c.]

**Figure 3:**
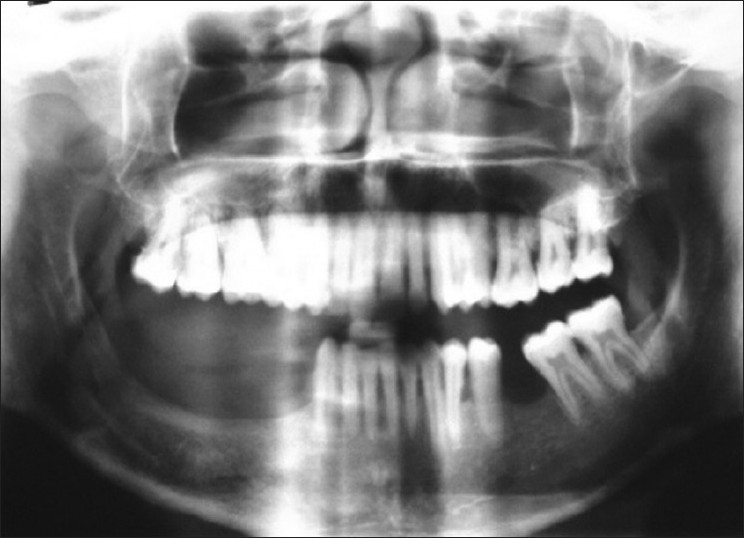
Postoperative panoramic radiograph, 1 year after curettage, showing no recurrence

A panoramic radiograph was taken after 1 year of follow-up, which revealed complete healing of the involved site with no evidence of recurrence [Figure 3].

## DISCUSSION

The term “aneurysmatic” refers to the “blow-out” effect or expansion of the affected bone that appears in these types of lesions.[9] The ABC of the jaw is a psuedocyst lacking epithelial lining.[6,9] It comprises 5% of all the lesions of the cranial and maxillofacial bones[3] and is most common in those regions of the skeleton where there is both a relatively high venous and marrow content. This explains the rarity of ABC in the skull bones, in which there is low venous pressure.[5]

The etiology of ABC is controversial. According to Steiner and Kantor, the ABC can develop as either a primary or secondary lesion associated with other bone diseases. Levy *et al*. had proposed that a history of trauma and subperiosteal hematoma formation is an essential factor in the development of ABC. Struthers and Shear have also concluded that ABC can occur as a secondary phenomenon in a pre-existing lesion and that central giant cell granuloma appears to be the most common of these lesions.[1] Tillman *et al*. have reported 95 cases with no history of trauma.[8] In the present case also, there was no history of trauma. Jaffe and Lichentenstein refer to alterations in local hemodynamics causing increased venous pressures and engorgement of the vascular bed in the transformed bone, leading to resorption, connective tissue replacement and osteoid formation.[1,8] Hernandez *et al*. classified ABC as primary and secondary. Primary could be congenital or acquired and could originate from pre-existing AV malformations. The congenital type is seen in children and young adults with no history of trauma, whereas the acquired type is found in adults with a history of trauma. The secondary type is postulated to be associated with degeneration of pre-existing lesions such as a cyst, tumor or fibrosseous lesion.[1] The two lesions could exist independently. Hence, ABC is considered as nonneoplastic, fibrodysplastic, noncystic bone entity.[1,10] In the present case as no history of trauma was reported, the etiology could be either due to alterations in local hemodynamics or degeneration of any pre-existing lesion at the involved site. Panoutsakopoulos *et al*. had described three cases of ABC with chromosomal anomalies, involving band 16q22.[11] Familial incidence of ABC has also been reported in literature.[12–14]

ABCs are most commonly found in long bones and vertebral column; 1.9% are reported to occur in jaws. An unusual location for ABC, i.e., mandibular condyle and coronoid process has also been reported.[8,9,15] ABC is extremely variable in clinical presentation, ranging from a small, indolent, asymptomatic lesion to rapidly growing, expansile, destructive lesion causing pain, swelling, deformity, neurologic symptoms, pathologic fracture and perforation of the cortex.[3]

The radiological features of ABC in the jaws are quite conflicting; the bone is expanded, appears cystic resembling a honeycomb or soap bubble and is eccentrically ballooned. There may be destruction or perforation of the cortex and a periosteal reaction may be evident.[6] It may appear radiolucent, radiopaque or mixed. In our case, a unilocular radiolucency causing expansion of the cortical plates and thinning of the lower border of the mandible with root resorption of the involved teeth was present. The diagnosis based on radiographic appearance is impossible because there are other lesions having similar radiographic appearance, such as ameloblastoma, myxoma, central giant cell granuloma, odontogenic cysts or central hemangiomas of the bone.[16]

Histologically, ABC consists of many sinusoidal blood-filled spaces set in a fibrous stroma, with multinucleated giant cells and osteoid. Hemosiderin is present in variable amounts and there is evidence of osteoid and bone formation. This description is characteristic of the “classic or vascular” form.[9] The histologic features in our case were consistent with the above-mentioned features. Solid form is the other histological type, which is a noncystic variant with solid gray-white tissue, hemorrhagic foci and abundant fibroblastic and fibrohistiocytic elements with osteoclast-like giant cells, osteoblastic differentiation areas with osteoid and calcifying fibromyxoid tissue. The mixed form demonstrates elements of both vascular and solid types.[9]

Treatment of ABC is usually directed toward complete removal of the lesion. This may prove difficult at times since the lesions are often multilocular and may be divided by multiple bony septae.[3] The treatment modalities are percutaneous sclerotherapy, diagnostic and therapeutic embolization, curettage, block resection and reconstruction, radiotherapy and systemic calcitonin therapy.[5] Self-healing cases have also been reported on long-term follow-up.[17] Several authors recommend immediate reconstruction of the defect with autogenous grafts in cases of esthetic deformity, high risk of fractures and loss of mandibular continuity.[1,6,15] Simple curettage is associated with high recurrence rates varying from 21 to 50%. But Motamedi *et al*.[3] have reported that initial resection is not necessary and have not noted any recurrences following surgical curettage of mandibular lesions. The present case was treated by curettage and regularly monitored. There was no evidence of any residual lesion after 1 year of follow-up.

## CONCLUSION

As the radiological features of ABC are varied, resembling many lesions, histopathologic analysis is a must for the diagnosis.

## References

[1] Gadre KS, Zubairy RA (2000). Aneurysmal bone cyst of the mandibular condyle: Report of a case. J Oral Maxillofac Surg.

[2] Motamedi MH, Stavropoulos M.F (1997). Large radiolucent lesion of the mandibular condyle. J Oral Maxillofac Surg.

[3] Kalantar Motamedi MH (1998). Aneurysmal bone cyst of the jaws: Clinicopathological features, radiographic evaluation and treatment analysis of 17 cases. J Craniomaxillofac Surg.

[4] Rosenberg AE, Nielsen GP, Fletcher JA (2002). World Health Organisation Classification of tumours. Pathology and Genetics of Tumours of Soft tissues and Bone.

[5] Goyal A, Tyagi I, Syal R, Agrawal T, Jain M (2006). Primary aneurismal bone cyst of coronoid process. BMC Ear Nose Throat Disord.

[6] Kiattavorncharoen S, Joos U, Brinkschmidt C, Werkmeister R (2003). Aneurysmal bone cyst of the mandible: A case report. Int J Oral Maxillofac Surg.

[7] López-Arcas JM, Cebrián L, González J, Burgueño M (2007). Aneurysmal bone cyst of the mandible: Case presentation and review of the literatura. Med Oral Patol Oral Cir Bucal.

[8] Pelo S, Gasparini G, Boniello R, Moro A, Amoroso PF (2009). Aneurysmal bone cyst located in the mandibular condyle. Head Face Med.

[9] Capote-Moreno A, Acero J, García-Recuero I, Ruiz J, Serrano R, de Paz V (2009). Giant aneurysmal bone cyst of the mandible with unusual presentation. Med Oral Patol Oral Cir Bucal.

[10] Eisenbud L, Attie J, Garlick J, Platt N (1987). Aneurysmal bone cyst of the mandible. Oral Surg Oral Med Oral Pathol.

[11] Panoutsakopoulos G, Pandis N, Kyriazoglou I, Gustafson P, Mertens F, Mandahl N (1999). Recurrent t(16; 17)(q22; p13) in aneurismal bone cysts. Genes Chromosom Cancer.

[12] Vicenzi G (1981). Familial incidence in two cases of aneurysmal bone cyst. Ital J Orthop Traumatol.

[13] Power RA, Robbins PD, Wood DJ (1996). Aneurysmal bone cyst in monozygotic twins: A case report. J Bone Joint Surg Br.

[14] Leithner A, Windhager R, Kainberger F, Lang S (1998). A case of aneurysmal bone cyst in father and son. Eur J Radiol.

[15] Martins WD, Fávaro DM (2005). Aneurysmal bone cyst of the coronoid process of the mandible: A case report. J Contemp Dent Pract.

[16] Karabouta I, Tsodoulos S, Trigonidis G (1991). Extensive aneurysmal bone cyst of the mandible: Surgical resection and immediate reconstruction. Oral Surg Oral Med Oral Pathol.

[17] Malghem J, Maldague B, Esselinck XW, Noel H, De Nayer P, Vincent A (1989). Spontaneous healing of aneurysmal bone cysts: A report of three cases. J Bone Joint Surg Br.

